# Supplementary material for: A novel quantification method for retinal pigment epithelium phagocytosis using a very-long-chain polyunsaturated fatty acids-based strategy
Source: Front Mol Neurosci. 2023 Oct 20;16:1279457. doi: 10.3389/fnmol.2023.1279457 (PMC10622967; doi:10.3389/fnmol.2023.1279457)
Supplement: Supplementary file 1 [file Presentation_1.pdf]

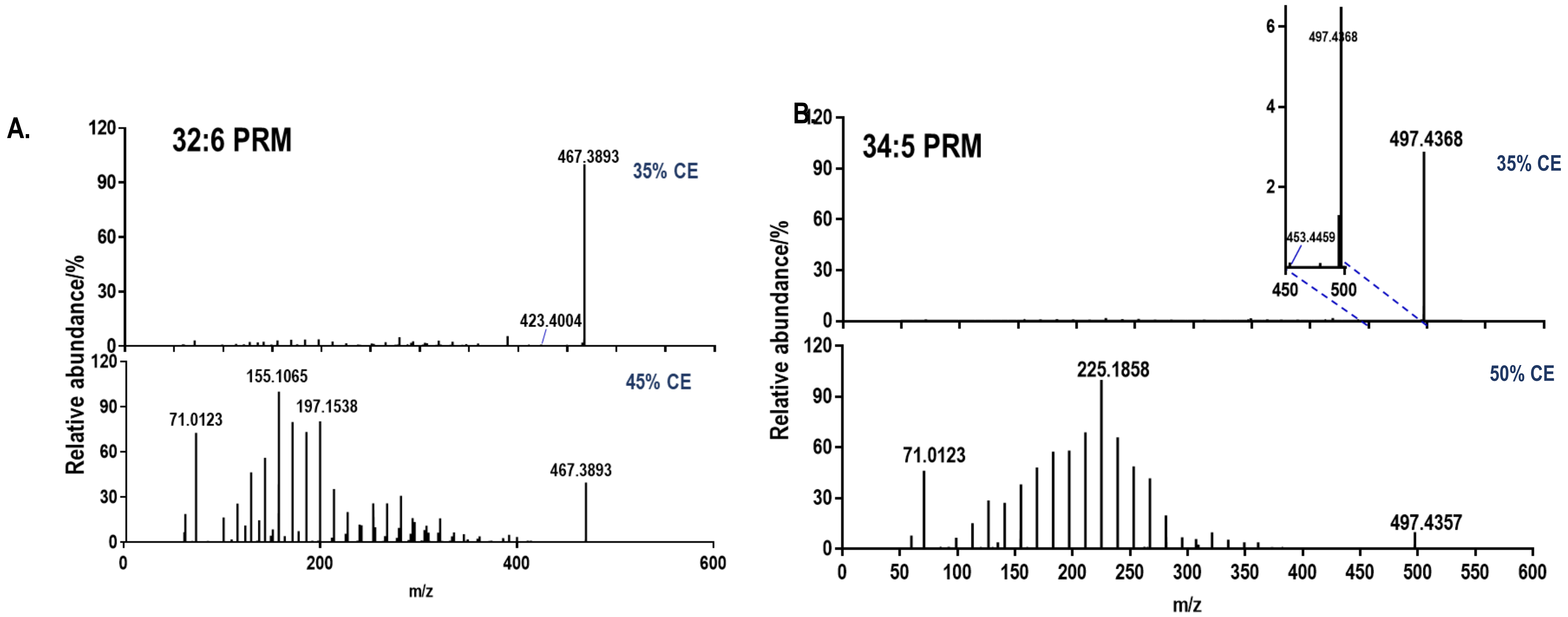


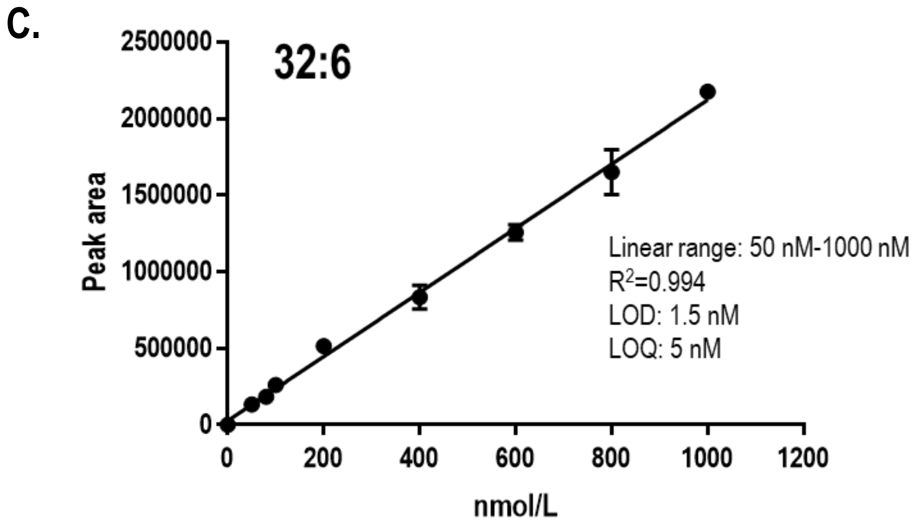


D.

| VLC-PUFAs | SD%(n=5) |
|---|---|
| 32:6 | 7.14 |
| 34:4 | 14.59 |
| 34:5 | 6.89 |
| 34:6 | 6.46 |
| 36:6 | 9.42 |

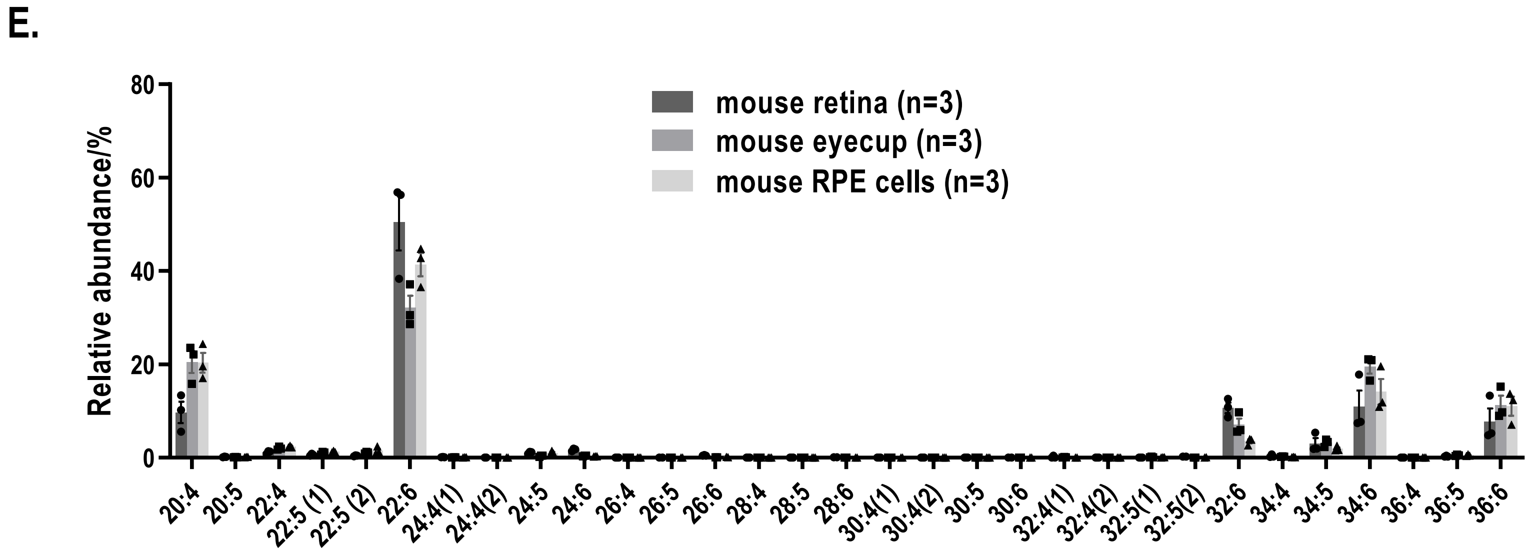


**Supplementary Figure 1. Identification and quantification of VLC-PUFAs by LC-MS and LC-MS/MS method.**

Peak clusters with a mass difference of 14 Da corresponding to $CH_2$ in parallel reaction monitoring (PRM) fragment ion chromatograms of 32: 6 **(A)** and 34:4 **(B)** from bovine retina extract.

**C.** Linearity and linear range for 32:6 in FS mode. Linear correlation coefficient = 0.994 from 50 nmol/L to 1000 nmol/L, and the limit of quantification of 32:6 = 5 nmol/L (n=5).

**D.** The standard deviations of the peak area of 5 VLC-PUFAs from bovine retina extract were below 15% (n=5).

**E.** Abundance plots present the relative abundances within the total abundance of all the detected LC-PUFAs and VLC-PUFAs in mouse samples. LC-PUFAs and VLC-PUFAs composition in mouse retina, eyecup and RPE cells exhibited high abundance of VLC-PUFAs, ranging from 32:6 to 36:6. n represented number of retinas and eyecups was used, while in mouse RPE cells sample, n represented number of mice (2 eyecups/mice). Retinas and eyecups were collected in the morning.

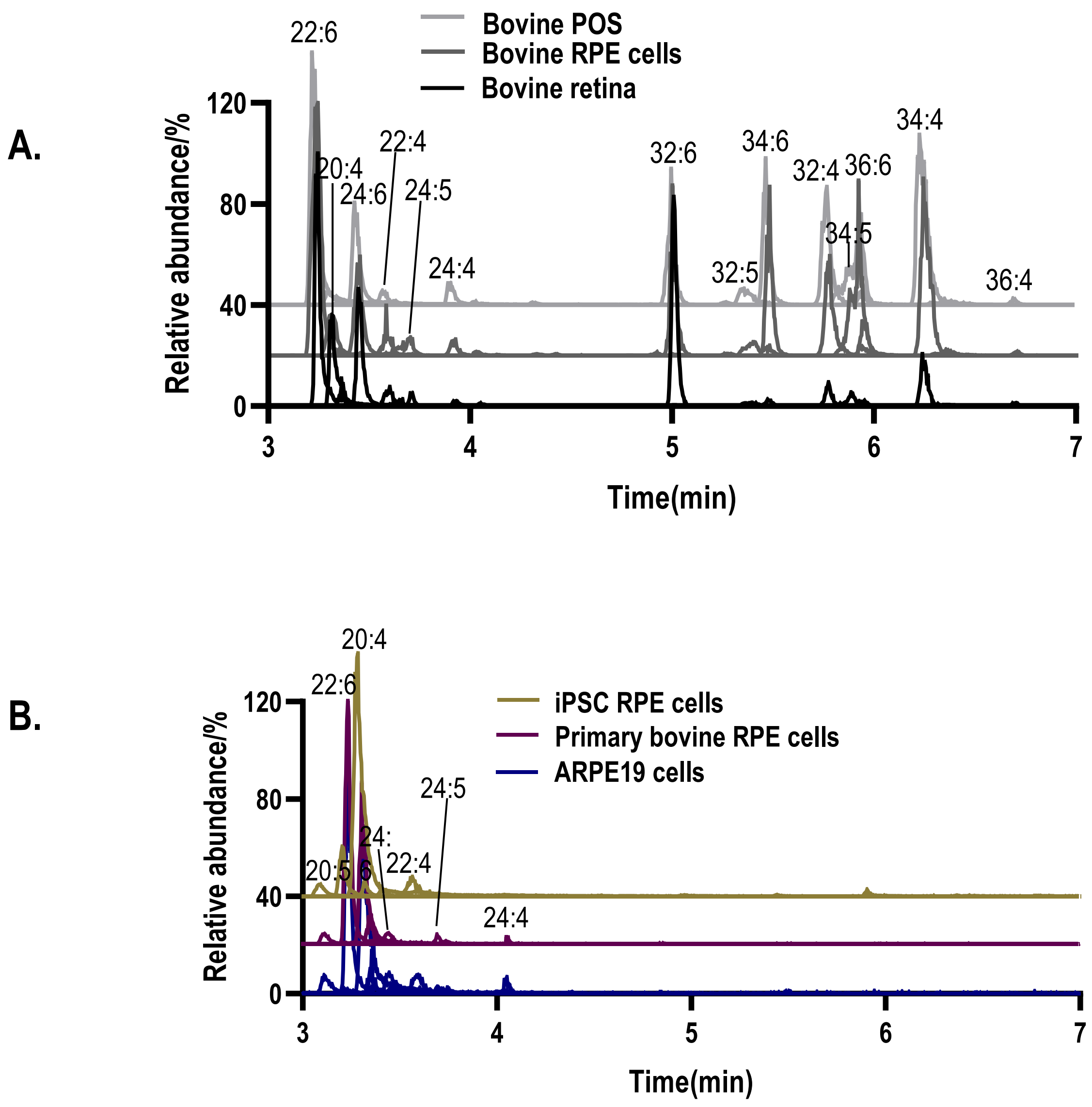


**Supplementary Figure 2. Extracted ion chromatograms (EIC) of LC-PUFAs and VLC-PUFAs.**

**A.** EIC of LC-PUFAs and VLC-PUFAs extracted from bovine retina, bovine RPE cells and bovine POS.

**B.** EIC of LC-PUFAs and VLC-PUFAs extracted from ARPE19 cells, primary bovine RPE cells and iPSC-RPE cells.

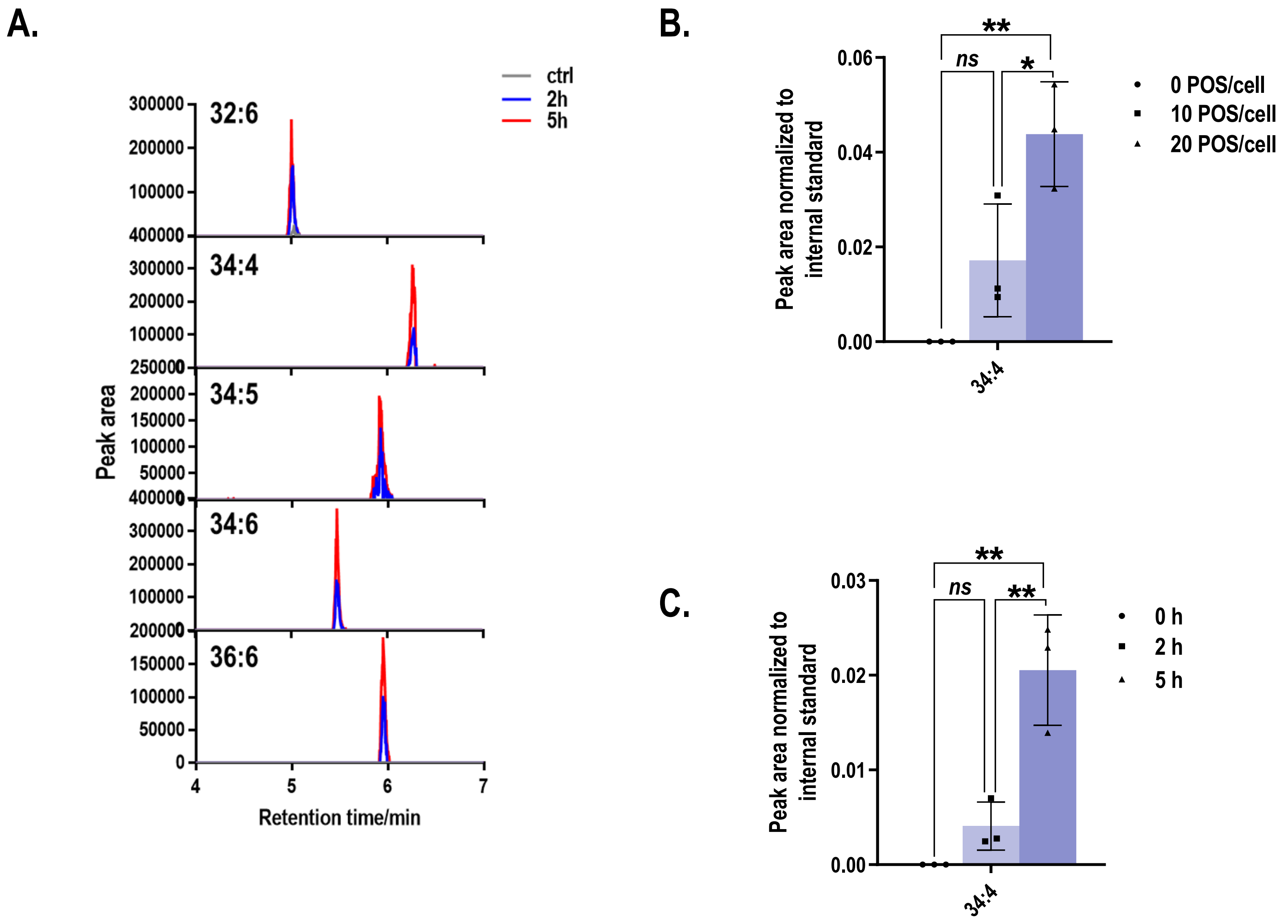


**Supplementary Figure 3. VLC-PUFAs-based quantification can detect gradual changes in phagocytosis**

**A.** Extracted ion (EIC) mass chromatograms of marker VLC-PUFAs obtained in FS mode by LC–ESI(-)–MS (FS) after incubating with 20 POS/cell for 2 and 5 hours.

Quantification of phagocytosis was performed by using 34:4 as one of POS marker VLC-PUFAs when ARPE-19 cells were challenged with increasing POS concentrations **(B)** or increasing incubation times **(C)**. (n=3, * = p<0.05, ** = p<0.01)
